# Decreased heart rate variability responses during early postoperative mobilization – an observational study

**DOI:** 10.1186/s12871-015-0099-4

**Published:** 2015-08-22

**Authors:** Øivind Jans, Louise Brinth, Henrik Kehlet, Jesper Mehlsen

**Affiliations:** 1 Section of Surgical Pathophysiology, Copenhagen University Hospital, Rigshospitalet, Blegdamsvej 9, DK-2100 Copenhagen, Denmark; 2 The Lundbeck Centre for Fast-track Hip and Knee Arthroplasty, Rigshospitalet, Copenhagen, Denmark; 3 Coordinating Research Centre, Frederiksberg Hospital, Frederiksberg, Denmark

## Abstract

**Background:**

Intact orthostatic blood pressure regulation is essential for early mobilization after surgery. However, postoperative orthostatic hypotension and intolerance (OI) may delay early ambulation. The mechanisms of postoperative OI include impaired vasopressor responses relating to postoperative autonomic dysfunction. Thus, based on a previous study on haemodynamic responses during mobilization before and after elective total hip arthroplasty (THA), we performed secondary analyses of heart rate variability (HRV) and aimed to identify possible abnormal postoperative autonomic responses in relation to postural change.

**Methods:**

A standardized mobilization protocol before, 6 and 24 h after surgery was performed in 23 patients scheduled for elective THA. Beat-to-beat arterial blood pressure was measured by photoplethysmography and HRV was derived from pulse wave interbeat intervals and analysed in the time and frequency domain as well as by non-linear analysis using sample entropy

**Results:**

Before surgery, arterial pressures and HR increased upon standing, while HRV low (LF) and high frequency (HF) components remained unchanged. At 6 and 24 h after surgery, resting total HRV power, sample entropy and postural responses in arterial pressures decreased compared to preoperative conditions. During standing HF variation increased by 16.7 (95 % CI 8.0–25.0) normalized units (nu) at 6 h and 10.7 (2.0–19.4) nu at 24 h compared to the preoperative evaluation. At 24 h the LF/HF ratio decreased from 1.8 (1.2–2.6) nu when supine to 1.2 (0.8–1.8) nu when standing.

**Conclusions:**

This study observed postoperative autonomic cardiovascular dysregulation that may contribute to limited HRV responses during early postoperative mobilization.

**Trial registration:**

ClinicalTrials.gov NCT01089946

## Background

An intact postural blood pressure regulation after surgery is essential for early postoperative mobilization and rapid functional recovery [1]. However, early mobilization, often instituted on the same day as the surgical procedure, may be delayed by postoperative orthostatic-hypotension (OH) and intolerance (OI) which is characterized by symptoms such as dizziness, nausea, blurred vision or even syncope during postoperative mobilization [2]. Transient inability to ambulate has even been observed after planned ambulatory surgery and may therefore contribute to prolonged hospital stay [3–5]. Few studies have evaluated OI during early postoperative mobilization, but the incidence of OI has been reported to be as high as 50 % in patients undergoing radical prostatectomy and in patients undergoing elective unilateral hip arthroplasty (THA) [6–8]. Postoperative OI has been evaluated by either tilt-table testing [9], or by a standardized mobilization protocol including continuous blood pressure measurements and mimicking the steps normally employed when mobilizing patients early after surgery, e.g. incorporating a few minutes of sitting bedside rest before transition to the upright position [6, 7, 10].

The pathophysiology of OI is not clearly understood but may be related to impaired cardiovascular regulation postoperatively. Thus, studies in both prostatectomy and THA patients found impaired postoperative postural vasopressor responses causing OH and cerebral deoxygenation in OI patients [6–8]. Furthermore, impairment in postoperative postural cardiovascular responses including impaired vasoconstriction was reported even in patients tolerant to the postoperative mobilization procedure. These findings suggest a general dysfunction in autonomic cardiovascular control in the early postoperative period that may relate to either defects in the baroreflex arc, the central integration of autonomic reflexes, or in the end organ response.

Analyses of heart rate variability (HRV) are widely used in assessing autonomous nervous system and decreased HRV indices have previously been reported after major abdominal surgery [11, 12]. However, these studies did not evaluate HRV in relation to early postoperative postural cardiovascular regulation.

Thus, based on data from a previous study evaluating cardiovascular responses to early mobilization after THA [7], we performed an analysis of heart rate variability derived from interbeat pulse intervals before and after surgery in patients scheduled for elective THA. We aimed to identify and characterize possible abnormal autonomic postural responses that could contribute to the pathophysiology of postoperative impaired arterial pressure regulation.

## Methods

This study was carried out based on heart rate data obtained from arterial pressure curves in a previous study evaluating the occurrence of OI in patients > 18 years undergoing unilateral primary THA in a standardized fast-track setting [7].

Exclusion criteria were history of OI, diabetes mellitus, atrial fibrillation, ASA-score ≥ 3 or a history of alcohol abuse (>40 units week-1).

Informed consent was obtained from all individual participants included in the study, which was approved by the local ethics committee (H-D-2009-067) and registered by the Danish data protections agency and on ClinicalTrials.gov under the US national library of medicine (NCT01089946).

### Anaesthesia and pain management

Patients were anaesthetized with spinal anaesthesia (12.5–15 mg bupivacaine) and received intraoperative propofol sedation at the discretion of the attending anaesthesiologist.

To cover basal and surgical fluid losses, a liberal fixed volume fluid regimen of 12 ml kg-1 isotonic saline was administered during the first hour of surgery followed by 6 ml kg-1 h-1 until end of surgery and 2 ml kg h-1 for the first 6 h after surgery with no restriction on postoperative oral fluid intake [13]. Intraoperative blood loss was replaced 1:1 with 6 % hydroxyl ethyl starch (Voluven; 130/0.4 Fresenius Kabi AB, Uppsala, Sweden).

Perioperative pain management was standardized as follows: slow-release acetaminophen 2 g and gabapentin 600 mg before surgery, continuing with 300 and 600 mg gabapentin at 8 am and 6 pm respectively and 2 g slow-release acetaminophen twice daily for the duration of hospital stay. During the first 24 h after surgery, high volume local infiltration analgesia with 150 ml ropivacaine 0.2 % was administered intraoperatively and 50 ml was administered 8 and 24 h after surgery. Pain scores were graded on a verbal rating scale (0–10) and if they exceeded 3 at rest or 5 during movement, patients received supplemental oxycodone.

### Orthostatic challenge

A standardized mobilization procedure was performed approximately 1 h before surgery and was repeated 6 and 24 h after the operation, defined from the time of wound closure. Mobilization included supine rest (5 min), followed by 30° passive leg raise (3 min), supine rest (5 min), sitting on the bed with the feet on the floor (3 min), followed by standing while the patient was verbally encouraged to stand on the toes and shift body weight from one leg to the other in order to activate the muscle pump and attenuate venous pooling in the legs (3 min) [14]. The mobilization procedure ended with recovery in the supine position (5 min) [6, 7]. The procedure was terminated if the patients reported symptoms of OI (dizziness, nausea, blurred vision) or if systolic arterial pressure (SAP) decreased more than 30 mmHg. Arterial blood pressure was measured at heart level on a beat-to-beat basis by photoplethysmography using a finger cuff applied on the middle part of the third finger (Nexfin®, BMeye, Amsterdam, The Netherlands) [15]. Interbeat intervals were derived from successive pulse upstrokes on the arterial blood pressure curve. During each postoperative mobilization test, fluid status and haemoglobin (Hb) were recorded and pain was graded for each body position. Before the 6 h test, remaining motor blockade was ruled out using a modified Bromage scale [16].

### Analysis of heart-rate variability

Arterial pressure curves were analysed off-line using the Nexfin@PC 1.0 software package (BMeye, Amsterdam, The Netherlands). Each curve was visually inspected for artefacts, and such data were excluded.

Interbeat-interval data derived from the arterial pressure curve were analysed in 3 min intervals for the following periods: Supine rest, sitting and standing. If 3 min sampling intervals were not available at baseline (supine), the patient was excluded entirely from the study and if 3 min sampling intervals in the sitting or standing positions were not obtainable due to OI or artefacts, data for that particular patient and time point were excluded.

Analysis of heart rate variability (HRV) was performed according to current recommendations using an evaluated share-ware (Kubios, vers. 2.0, http://kubios.uef.fi/) [12]. Before analysis, slow non-stationarities were removed by smoothness priors regularization [17]. Variations in interbeat-intervals in the time domain were quantified by mean values (meanNN) and standard deviations (SDNN) of normal interbeat-intervals in the supine, sitting and upright positions as well as by the standard deviation of the difference between successive normal beats (root mean successive squared difference, RMSSD). Prior to frequency analysis, interbeat-intervals were interpolated using cubic splines in order to allow for equidistant sampling at 4 Hz. The analysis was then performed by the autoregressive model using an order of 16 [18]. Total power (TP: 0.00–0.50 Hz) was calculated and low and high frequency components were derived from the 0.04–0.15 Hz band (LF) and from the 0.15–0.40 Hz band (HF), respectively and were expressed by absolute power (ms^2^) together with normalized units (nu) according to current guidelines [12]. The time and frequency domain analyses were supplemented by non-linear analysis expressed by the sample entropy.

### Statistical analyses

The underlying distribution for all variables was evaluated for normality and variables with a non-normal distribution (TP, LF power, HF power and the LF/HF ratio) were transformed with the natural logarithm in order to obtain normality prior to analysis. Differences in arterial pressure and HRV values within- (supine, sitting and standing) and between (preoperative, 6 and 24 h) mobilization sessions were evaluated using a mixed model ANOVA for repeated measures and compared to control levels (preoperative evaluation and supine position) using Dunnett’s Post-Hoc test. Preoperative TP and LF/HF ratio in the supine, sitting and standing position were compared between OI patients and patients tolerant to the mobilization procedure at 6 h after surgery using the independent samples *t*-test. Normally distributed data are presented as mean (±SD) with differences reported as mean (95 % CI). Transformed data are presented as geometric mean (95 % CI) or median (interquartile range). Statistical analyses were performed using SAS 9.2 (SAS Institute Inc., Cary, NC, USA) with a *P*-value of < 0.05 representing statistical significance.

## Results

The original dataset included 26 patients (17 women). However, the arterial pressure curves from three patients were excluded due to either movement artifacts or transient atrial fibrillation which did not allow for baseline HRV analysis in continuous three minute intervals at all three time points (pre, 6 h and 24 h). Accordingly, 23 patients were included. During mobilization at 6 h, HRV data for three and seven patients were excluded when sitting and standing, respectively, due to immediate OI or excessive artifacts, leaving data for HRV analysis in 20 and 16 patients, respectively. Likewise, at 24 h, HRV data were available for 19 and 20 patients at sitting and standing, respectively.

Patients had a mean (SD) age of 64 (10) years, height of 169 (11) cm and weight of 80 (18) kg. Fifteen (65 %) were female. The median ASA score was two, and nine (39 %) patients received oral antihypertensives (diuretics three, Ca^+^-antagonist two, ACE-inhibitor two, combination therapy two). None of the patients had a history of neurological disease or previous OI [7].

### Mobilization, arterial pressure responses and orthostatic intolerance

All patients completed the preoperative mobilization procedure without OI while nine (39 %) and five (22 %) had OI and terminated the procedure prematurely during standing at 6 and 24 h after surgery, respectively. The cardiovascular response to mobilization before and after THA in the original 26 patients has been described in detail previously [7]. The arterial pressures from the 23 analyzed patients before, 6 and 24 h after surgery are summarized in Table 1 and below. During the preoperative evaluation, the systolic (SAP) blood pressure increased from supine to sitting and standing by mean (95 % CI) 12 (3–20) and 16 (9–24) mmHg, respectively, while diastolic DAP blood pressure increased by 7 (3–10) and 11 (8–14) mmHg, respectively. In contrast, at 6 h after surgery, SAP did not change from supine to sitting but decreased from supine to standing by 18 (6–29) mmHg while DAP did not change significantly. At 24 h after surgery neither SAP nor DAP changed significantly with change in posture. Both SAP and DAP differed between the measurement sessions and were decreased both at 6 and 24 h for all 3 positions (supine, sitting and standing) compared to the preoperative evaluation (*P* < 0.05; Table 1)

**Table 1:** Cardiovascular data during mobilization before, 6 and 24 h after total hip arthroplasty

	Preoperative			6 h			24 h		
	Supine	Sitting	Standing	Supine	Sitting	Standing	Supine	Sitting	Standing
No of patients	23	23	23	23	20	16	23	19	20
SAP (mmHg)	141 (17)	155 (19)*	161 (19)*	131 (17)	134 (22)^§^	113 (31)^*,§^	127 (15)^§^	132 (23)^§^	126 (23)^§^
DAP (mmHg)	72 (8)	80 (7)*	85 (6)*	66 (9)^§^	70 (9)^§^	62 (13)^*,§^	63 (8)^§^	67 (9)^§^	64 (10)^§^
HR (bpm)	71 (11)	75 (12)	80 (13)*	82 (13)^§^	86 (14)^*,§^	88 (14)^*,§^	84 (12)^§^	87 (12)^*,§^	94 (12)^*,§^

Data presented as mean (SD)

SAP; systolic arterial pressure, DAP; diastolic arterial pressure, HR; heart rate, mmHg; millimeter mercury, bpm, beats per minute

*Different from supine (*P* < 0.05), § Different from preoperative evaluation (*P* < 0.05)

### Heart rate variability

MeanNN was reduced progressively in the supine position from preoperative (baseline) values (863 ± 144 ms) to 6 h (756 ± 141 ms) and 24 h (734 ± 115 ms; *p* < 0.001) after surgery (Table 2). In the standing position, meanNN increased from preoperative baseline values (771 ± 128 ms) to 781 ± 177 ms 6 h after surgery and decreased to 711 ± 157 ms (*p* = 0.005) 24 h after surgery. SDNN was reduced progressively in the supine position from baseline (22.1 ± 9.6 ms) to 6 h (16.4 ± 7.4 ms) and 24 h (13.5 ± 4.7 ms; *p* < 0.001). RMSSD was reduced progressively in the supine position from baseline (20.4 ± 8.2 ms) to 6 h (15.7 ± 6.5 ms) and 24 h (12.7 ± 3.9 ms; *p* < 0.001). There was no significant difference in SDNN or RMSSD when comparing baseline with postoperative values in the standing position.

**Table 2:** Heart rate variability data during mobilization before, 6 and 24 h after total hip arthroplasty

	Preoperative			6 h			24 h		
	Supine	Sitting	Standing	Supine	Sitting	Standing	Supine	Sitting	Standing
No of patients	23	23	23	23	20	16	23	19	20
MeanNN (ms)	863 (144)	825 (136)	771 (128)^*^	756 (141)^§^	718 (133)^*^	781 (177)^*,§^	734 (115)^§^	702 (106)^*^	711 (157)^*,§^
SDNN (ms)	22.1 (9.6)	23.5 (8.8)	20.2 (8.6)	16.4 (7.4)^§^	17.1 (6.6)	14.5 (3.2)^§^	13.5 (4.7)^§^	15.3 (5.5)	13.6 (5.0)^§^
RMSSD (ms)	20.4 (8.2)	22.2 (8.5)	18.9 (7.5)	15.7 (6.5)^§^	18.3 (6.8)	17.1 (3.6)	12.7 (3.9)^§^	16.3 (6.2)	14.5 (5.0)
Total HRV power (ms^2^)									
Geometric mean (95 % CI)	373 (255–547)	424 (289–621)	277 (189–407)^*^	187 (126–277)^§^	193 (128–290)^§^	133 (88–202)^§^	116 (80–166)^§^	135 (91–199)^§^	119 (81–175)^§^
Median (IQR)	295 (150–895)	432 (209–946)	278 (149–636)	151 (104–483)	177 (87–495)	167 (72–198)	118 (61–274)	144 (64–317)	99 (55–235)
LF variation (ms^2^)									
Geometric mean (95 % CI)	194 (125–303)	220 (142–343)	150 (97–234)^*^	84 (51–137)^§^	78 (47–131)^§^	52 (31–89)^*,§^	62 (39–97)^§^	64 (40–103)^§^	53 (34–83)^§^
Median (IQR)	157 (69–488)	263 (93–605)	165 (73–383)	87 (31–300)	67 (38–206)	61 (26–108)	65 (28–159)	59 (29–120)	38 (25–123)
HF variation (ms^2^)									
Geometric mean (95 % CI)	112 (77–164)	120 (82–175)	75 (51–109)^*^	60 (43–86)^§^	70 (48–101)^§^	52 (35–77)	35 (26–48)^§^	54 (39–75)^*,§^	43 (31–60)^§^
Median (IQR)	90 (57–255)	111 (63–230)	77 (33–164)	63 (33–126)	59 (37–139)	60 (45–77)	37 (21–49)	52 (21–105)	36 (22–86)
Normalized HRV values									
LF variation (nu)	62.3 (14.8)	62.9 (16.8)	66.5 (13.1)	57.4 (20.6)	53.4 (20.6)^§^	48.4 (18.9)^§^	61.1 (18.8)	53.8 (19.2)^*,§^	55.9 (17.1)^*,§^
HF variation (nu)	37.7 (14.8)	37.1 (16.8)	33.5 (13.1)	42.6 (20.6)	46.6 (20.6)^§^	51.6 (18.9)^§^	38.9 (18.8)	46.2 (19.2)^*,§^	44.1 (17.1)^*,§^
LF/HF ratio									
Geometric mean (95 % CI)	1.7 (1.3**–**2.4)	1.8 (1.3**–**2.5)	2.0 (1.5**–**2.8)	1.4 (0.9**–**2.1)	1.1 (0.7**–**1.7)^§^	1.0 (0.7**–**1.6)^§^	1.8 (1.2**–**2.6)	1.2 (0.8**–**1.8)^*^	1.2 (0.8**–**1.8)^*,§^
Median (IQR)	1.7 (1.3**–**2.5)	2.0 (1.1**–**3.0)	2.1 (1.4**–**2.6)	1.5 (0.9**–**2.6)	1.1 (0.6**–**2.4)	1.0 (0.7**–**1.6)	1.7 (0.8**–**3.2)	1.2 (0.5**–**2.0)	1.1 (0.7**–**2.6)

Data presented as mean (SD) for normally distributed variables, or median (interquartile range) and geometric mean (95 % CI) for skewed data

mmHg; millimeter mercury, bpm; beats per minute, ms; milliseconds, nu; normalized units

*Different from supine (*P* < 0.05), § Different from preoperative evaluation (*P* < 0.05)

**Fig. 1:**
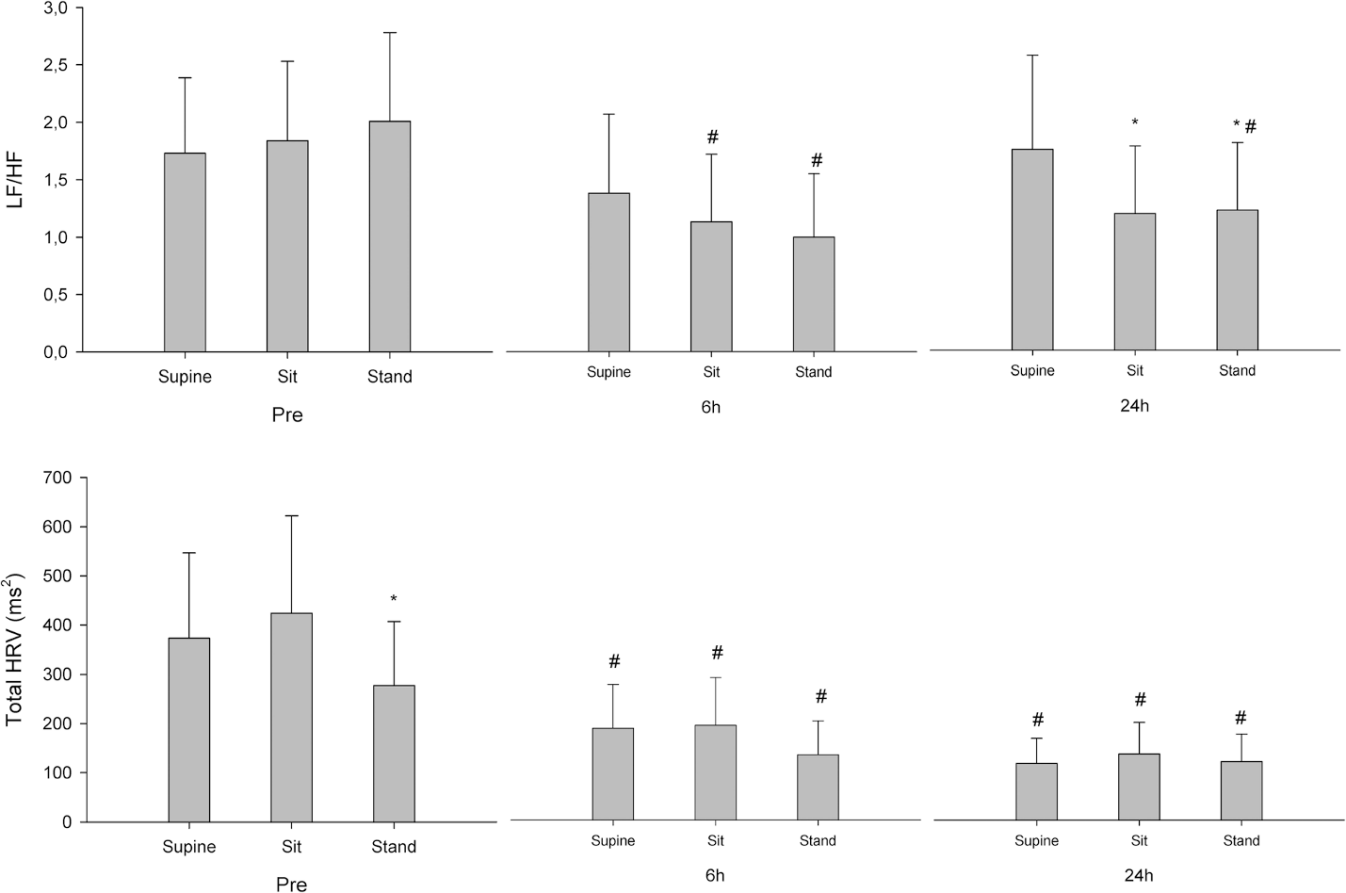
Total heart rate variability (HRV) and low to high frequency ratio (LF/HF) in THA patients during postural change before (pre), 6- and 24 h after surgery. Data represent geometric means with 95 % confidence intervals. # Different from preoperative evaluation (*P* < 0.05), *Different from supine (*P* < 0.05)

**Fig. 2:**
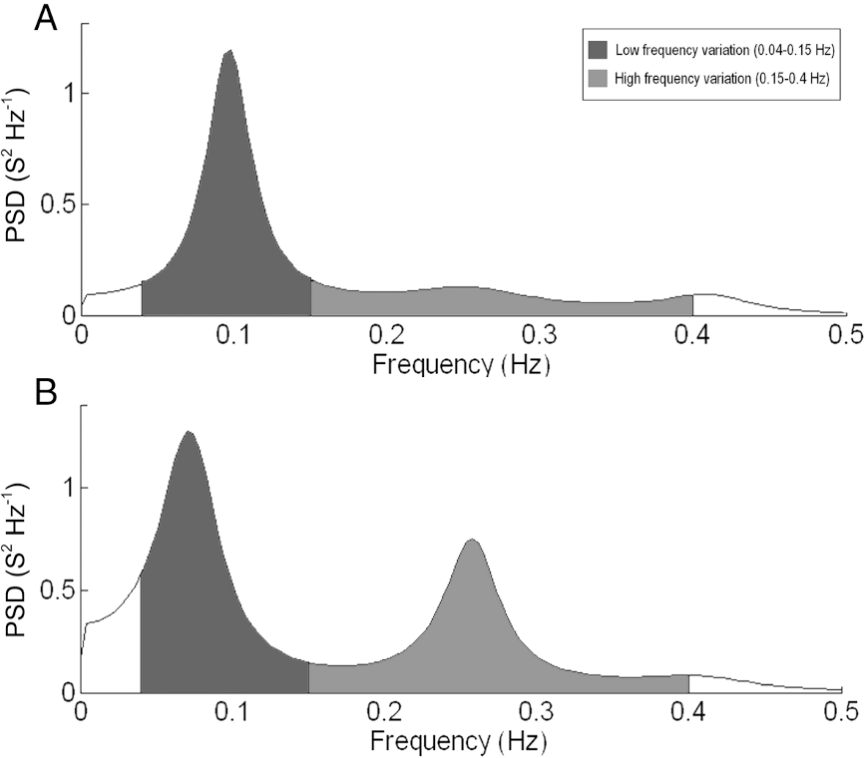
Autoregressive model of the frequency spectrum of heart rate variation before (panel A) and 6 h after surgery (panel B) for a single THA patient in the sitting position. Dark grey areas represent low frequency variation (0.04–0.15 Hz) and light grey areas represent high frequency variation (0.15–0.40 Hz). PSD; power spectral density, Hz, Hertz; s; seconds

Total power was reduced progressively in the supine position from baseline 373 (255–547 ms^2^), geometric mean (95 % CI), to 6 h 187 (126–277 ms^2^) and 24 h 116 (80–166 ms^2^; *p* < 0.001) postoperatively (Fig. 1). In the standing position, total power was reduced progressively from baseline 277 (189–407 ms^2^) to 6 h 133 (88–202 ms^2^) and 24 h 119 (81–175 ms^2^; *p* = 0.026) postoperatively. LF was unchanged from baseline in the supine position, but decreased during standing from baseline (66.5 ± 13.1 nu) to 6 h (48.4 ± 18.9 nu) and 24 h (55.9 ± 17.1 nu; *p* < 0.001). HF was unchanged in the supine position, but increased during standing from baseline (33.5 ± 13.1 nu) to 6 h (51.6 ± 18.9 nu) and 24 h (44.1 ± 17.1 nu; *p* < 0.001). At 24 h LF decreased with posture from supine (61.1 ± 18.8 nu) to standing (55.9 ± 17.1 nu; *p* < 0.05) with a concomitant increase in HF from (38.9 ± 18.8 nu) to (44.1 ± 17.1 nu). The resulting changes in the LF/HF ratio are presented in Table 2 and Fig. 1. Figure 2 displays the HRV frequency distribution during the preoperative evaluation and at 6 h in the sitting position for a single patient.

**Fig. 3:**
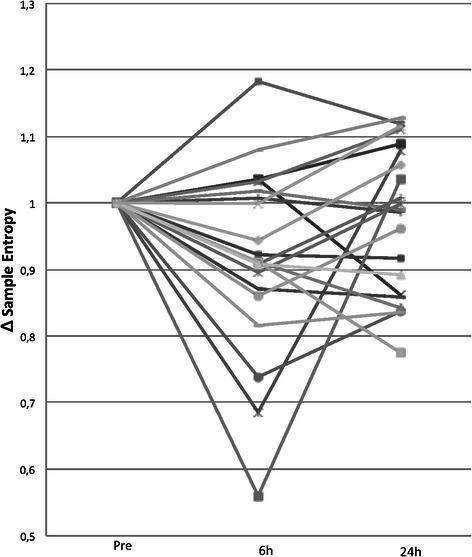
Relative changes in sample entropy for 23 patients in the supine position 6- and 24 h after surgery compared to the preoperative evaluation

There was no difference in the preoperative values of total power, LF, HF or the LF/HF ratio for all 3 body positions between patients completing the 6 h mobilization test and those terminating the test prematurely due to OI (Table 3). Figure 3 displays relative changes in sample entropy for all 23 patients compared to the preoperative evaluation. Sample entropy was reduced in the supine position from preoperative baseline values (1.83 ± 0.15) to 6 h (1.68 ± 0.24; *p* = 0.012) but returned to baseline values at 24 h (1,78 ± 0.22 ms^2^; n.s.).

**Table 3:** Total heart rate variability power and low to high frequency ratio during preoperative mobilization (n =23) grouped by orthostatic competence 6 h after surgery

	OT 6 h	OI 6 h	*P*-Value
Supine			
Total HRV power (ms^2^)	344 (203**–**585)	422 (201**–**855)	0.62
LF/HF ratio	1.5 (1.0**–**2.3)	2.2 (1.4**–**3.3)	0.22
Sitting			
Total HRV power (ms^2^)	397 (246**–**640)	471 (232**–**957)	0.65
LF/HF ratio	1.4 (0.9**–**2.2)	2.7 (1.5**–**4.9)	0.06
Standing			
Total HRV power (ms^2^)	258 (147**–**453)	326 (137**–**778)	0.60
LF/HF ratio	1.7 (1.2**–**2.5)	2.8 (1.7**–**4.8)	0.09

OT; Orthostatic tolerant, OI; Orthostatic intolerant, ms; milliseconds

Data presented as geometric mean (95 % CI)

## Discussion

The main findings of this first study of HRV data from patients during mobilization in the early postoperative phase after THA is the significant reduction in overall HRV and a paradoxically change in HRV towards the high frequency range during postural change from supine to standing.

A previous study in 30 patients undergoing abdominal surgery also found decreased HRV at the first postoperative day [11]. In this study, the HRV decrease correlated with both the duration of surgery and the amount of blood loss and thus HRV was proposed as method to evaluate surgical stress. In addition, a recent study in 30 elderly patients undergoing major non-cardiac surgery also reported decreased postoperative HRV compared to the preoperative evaluation [19]. These findings are in agreement with the overall decrease in postoperative HRV measures observed in our study. HRV during early postoperative mobilization has not previously been evaluated and we found a paradoxical postoperative shift in the frequency distribution with higher levels of HF variation and a decrease in the LF/HF ratio in the upright position which deviates from the normal response where LF/HF ratio is increased from the supine to standing position due to increased sympathetic and/or decreased vagal outflow [20, 21]. In addition to the observed changes in HRV, blood pressure responses were also attenuated during postoperative mobilization. In contrast to the preoperative evaluation, postural arterial pressure responses were impaired 6 and 24 h post-operatively, including an overall decrease in systolic blood pressure during standing 6 h after surgery.

Taken together, our findings suggest an impairment of baroreflex control during postural change in the early postoperative period, either centrally, by an attenuated sympathetic response or a relatively increased parasympathetic activity, or by peripheral impairment by delayed vascular reactivity.

The cause of abnormal postoperative blood pressure and HRV responses during postoperative mobilization is unclear but may be explained by several factors related to the surgical intervention. Obviously, postoperative hypovolaemia might have influenced postoperative orthostatic competence and the postural cardiovascular response. However, in order to prevent hypovolaemia, patients underwent a liberal intra- and postoperative fluid protocol and we sought to identify hypovolaemia prior to mobilization by a passive leg raise test. Furthermore, a recent randomized trial evaluating intra- and postoperative goal-directed fluid therapy (GDT) concluded that avoiding hypovolaemia by GDT was not sufficient to decrease the occurrence of OI during mobilization after prostatectomy [8]. Thus, we consider it unlikely that hypovolaemia contributed markedly to our findings. Although the patients received intraoperative propofol sedation, which may depress baroreflex sensitivity [22], the effect may be negligible during mobilization 6- and 24 h after surgery. This is also argued from our previous study in mastectomy where patients received propofol but without postural hemodynamic impairment [10]. Despite patients receiving a multimodal opioid-sparing analgesic regimen, opioids were administered as a rescue analgesic and median 9 and 17 mg oxycodone were administered 6 and 24 h after surgery, respectively. Although these doses were low, opioids are known to dampen efferent baroreflex activity in humans, but with differential effects on the sympathetic nerve activity to muscle and to the heart [23]. However, the vagotonic effects of opioid administration are well established [24], and thus, the use of opioids may have resulted in both a blunting of the baroreflex control and to a shift toward a higher frequency of HRV. However, it is also known that the efferent parasympathetic system is an integral part of the “neuroinflammatory” reflex acting as a negative feedback for cytokine production and as such a relative increase in vagal activity could be looked upon as normal response in the postoperative stage due to surgical inflammation [25]. Furthermore, a suppression of HRV and central downregulation of sympathetic vasomotor tone have been demonstrated in healthy volunteers during induction of systemic inflammation by lipopolysaccharide injection [26, 27]. Thus, the magnitude of the surgical procedure and the resulting inflammatory response may be of importance as impaired postural arterial pressure responses and OI were observed after major surgical procedures such as radical prostatectomy and total hip arthroplasty [6, 7], but not after breast cancer surgery in a study using the same mobilization protocol and methodology [10].

It may be argued that effects of the spinal anaesthesia might have contributed to our findings as impairment in vasomotor function due to residual sympathetic blockade might have shifted fluid towards capacitance vessels and have impaired the ability to vasoconstrict during postural change. However, impaired cardiovascular and HRV responses were also observed during the 24 h mobilization where any residual effects of spinal anaesthesia are absent. Also, similar postural hemodynamic changes to those observed in our study have been observed in prostatectomy without the use of neuraxial blockade [6].

Impaired haemodynamic- and HRV responses in the postoperative period may contribute to OH and OI during mobilization which have been demonstrated to prolong length of hospital stay after prostatectomy [8]. Furthermore, postoperative dizziness was a major reason for remaining hospitalized after TKA and THA [28]. Thus, exploratory studies like the present may contribute to elucidating the mechanisms behind OI and aid in future strategies for prevention. However, the present study is limited by the fact that it was a secondary analysis based upon data from a previous study, and thus the original sample size was calculated to show differences in the systolic blood pressure response and not HRV before and after surgery. In addition, due to the mobilization protocol, the interbeat intervals were obtained for a 3-min period in each body position, whereas established guidelines suggest recording for 5 min in short term HRV analyses. However, a 3-min sampling period exceeds the minimum period required (1–2 min) for obtaining reliable measures of LF and HF components [12]. Furthermore, due to immediate OI, a continuous 3-min RR-interval recording was not available during standing in 7 of 23 patients at 6 h after surgery. Although we hypothesized that impaired postoperative autonomic blood pressure regulation is a general defect related to surgery or anaesthesia, the reduced sample size at 6 h precluded analysis of whether orthostatic HRV responses could predict the occurrence of postoperative OI or whether HRV responses differed between OI patients and patients completing the mobilization procedure. Thus all available patients were included in an overall analysis of blood pressure responses and HRV before and after surgery. We used beat-to-beat pressure curves to derive pulse interbeat intervals for HRV analysis rather than RR-intervals derived from an ECG, which is considered the gold-standard [12]. However, pulse derived HRV have been compared to HRV derived from ECG in several studies and have demonstrated sufficient accuracy in subjects at rest [29–31], although short term variability such as the HF component may be overestimated with standing and exercise due to variations in pulse transit time [31]. An overestimation of the HF component during standing may have influenced our results, but an increase in HF during standing was not observed in the preoperative test and thus the postoperative postural decrease in the LF/HF ratio cannot be explained by the inherent limitations of pulse derived HRV analysis.

## Conclusions

In conclusion, this study observed postoperative autonomic cardiovascular dysregulation that may contribute to limited HRV responses during early postoperative mobilization.

## Abbreviations

ANOVA: Analysis of variance
BPM: Beats per minute
DAP: Diastolic arterial pressure
HF: High frequency
HRV: Heart rate variability
LF: Low frequency
MeanNN: Mean of interbeat intervals
mmHg: Millimetre mercury
MS: Milliseconds
NU: Normalised units
OH: Orthostatic hypotension
OI: Orthostatic intolerance
RMSSD: Root mean-squared successive differences between inter-beat intervals
SAP: Systolic arterial pressure
SDNN: Standard deviation of inter-beat intervals
THA: Total hip arthroplasty
TKA: Total knee arthroplasty
